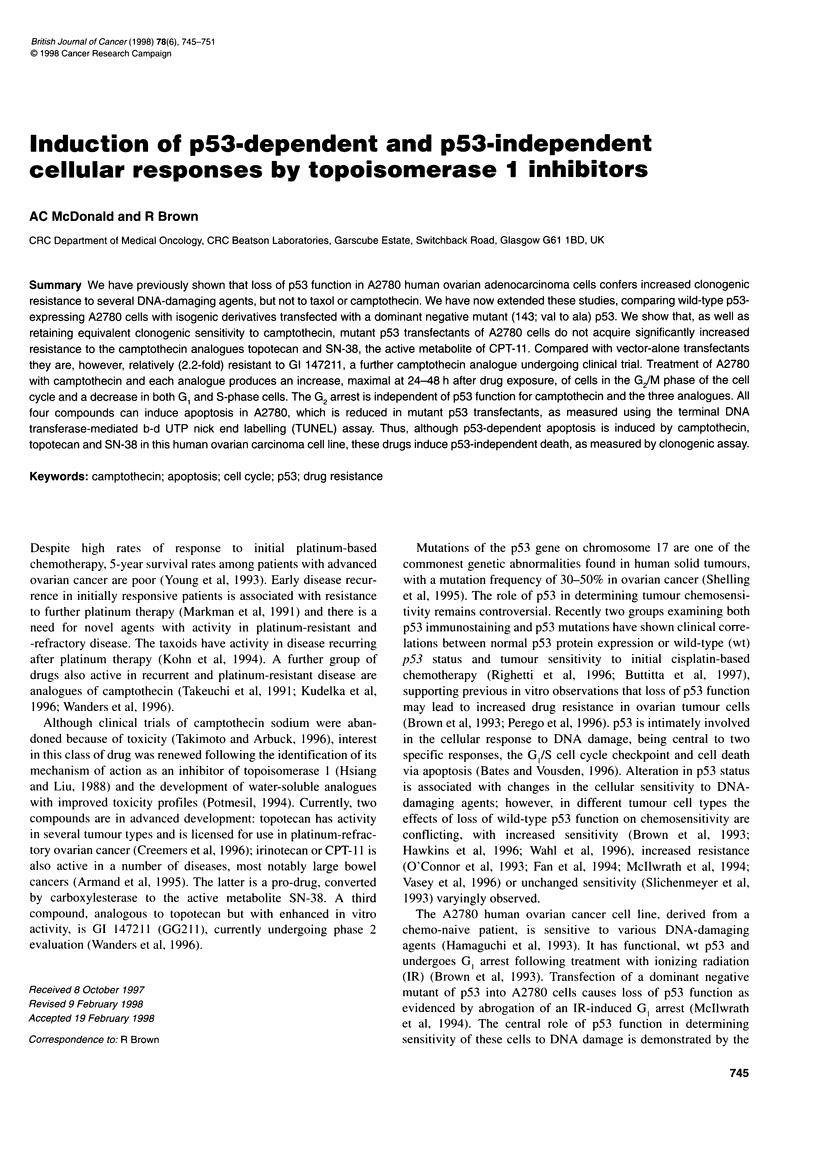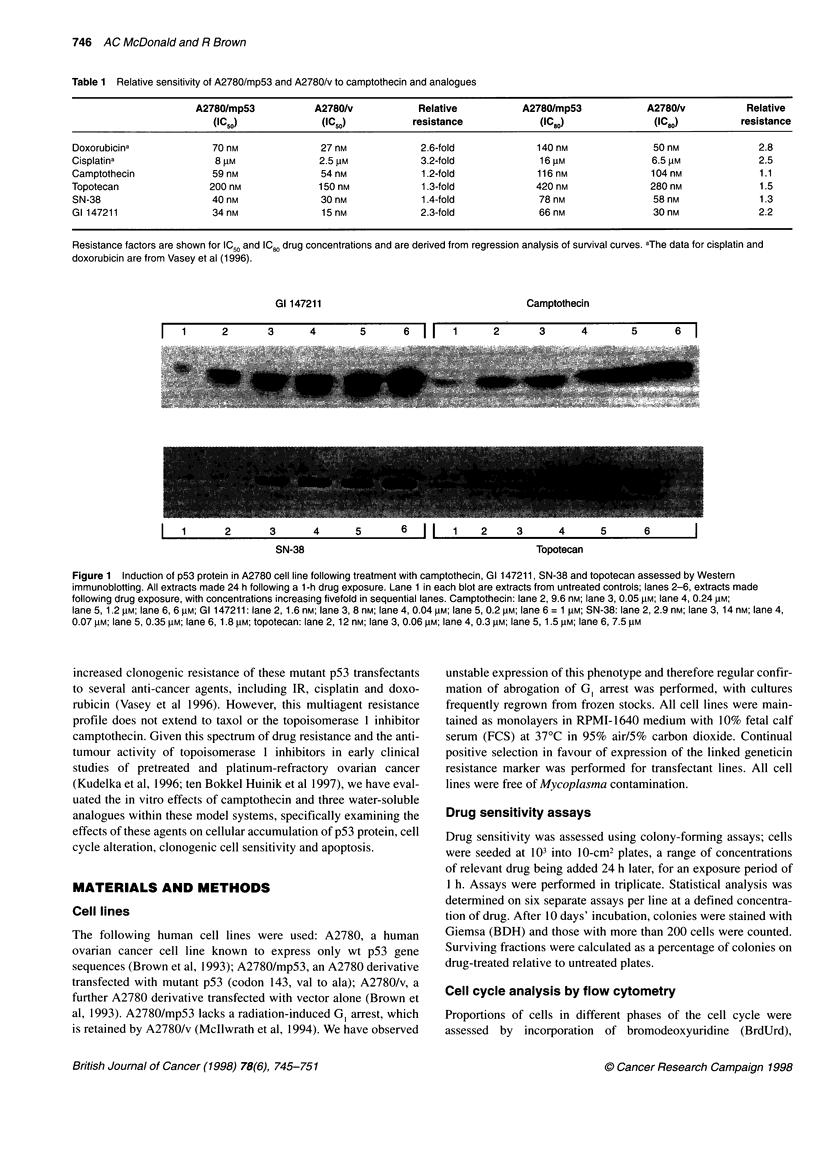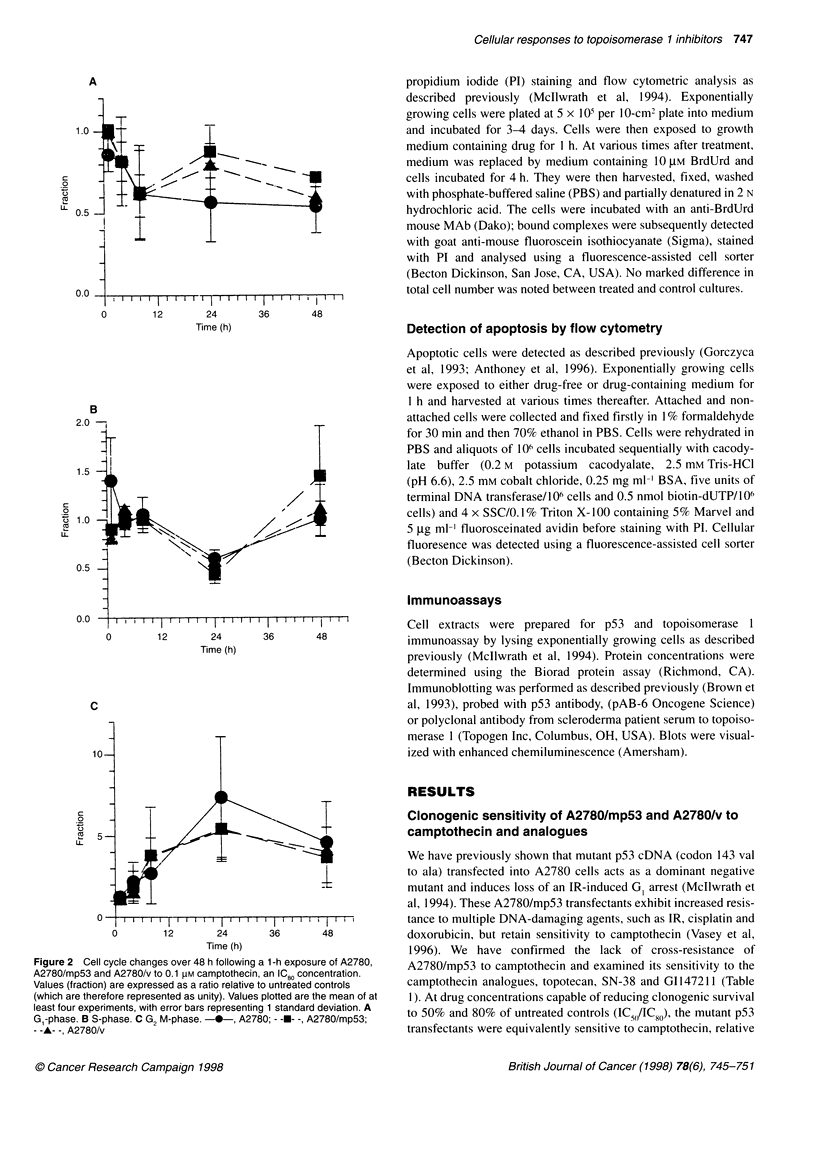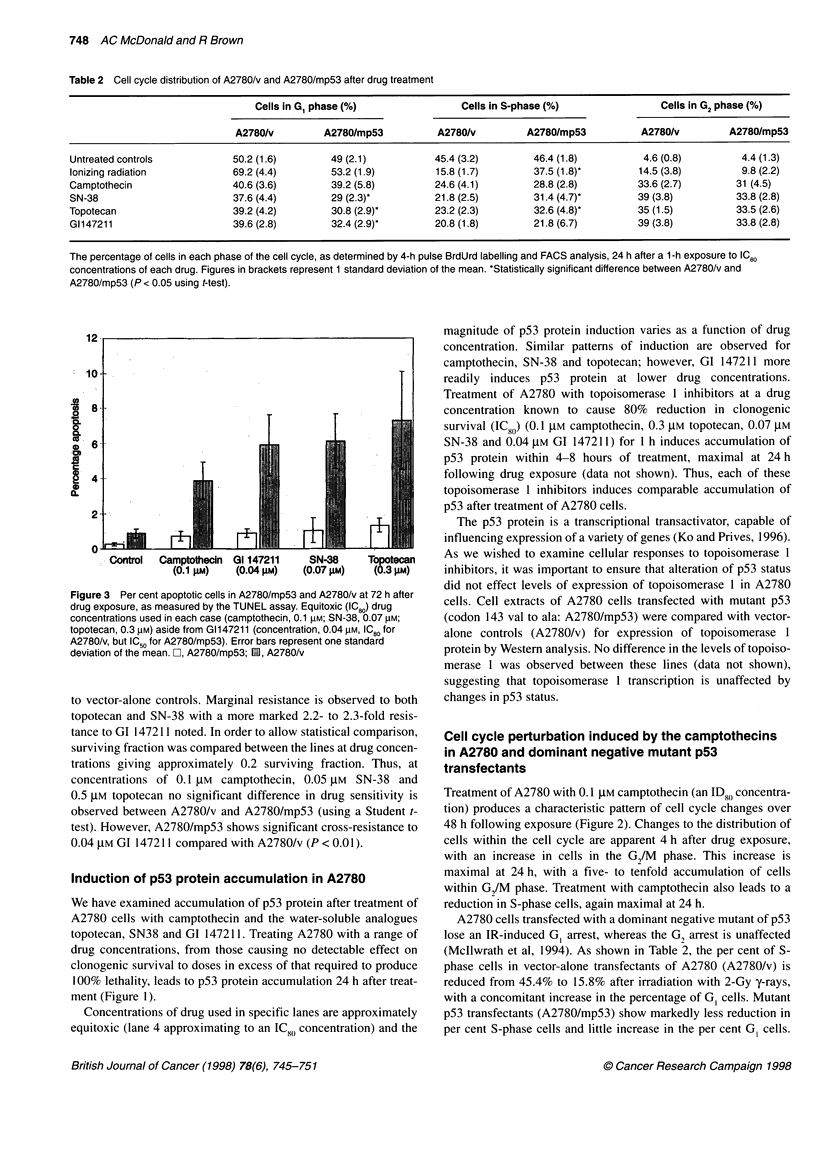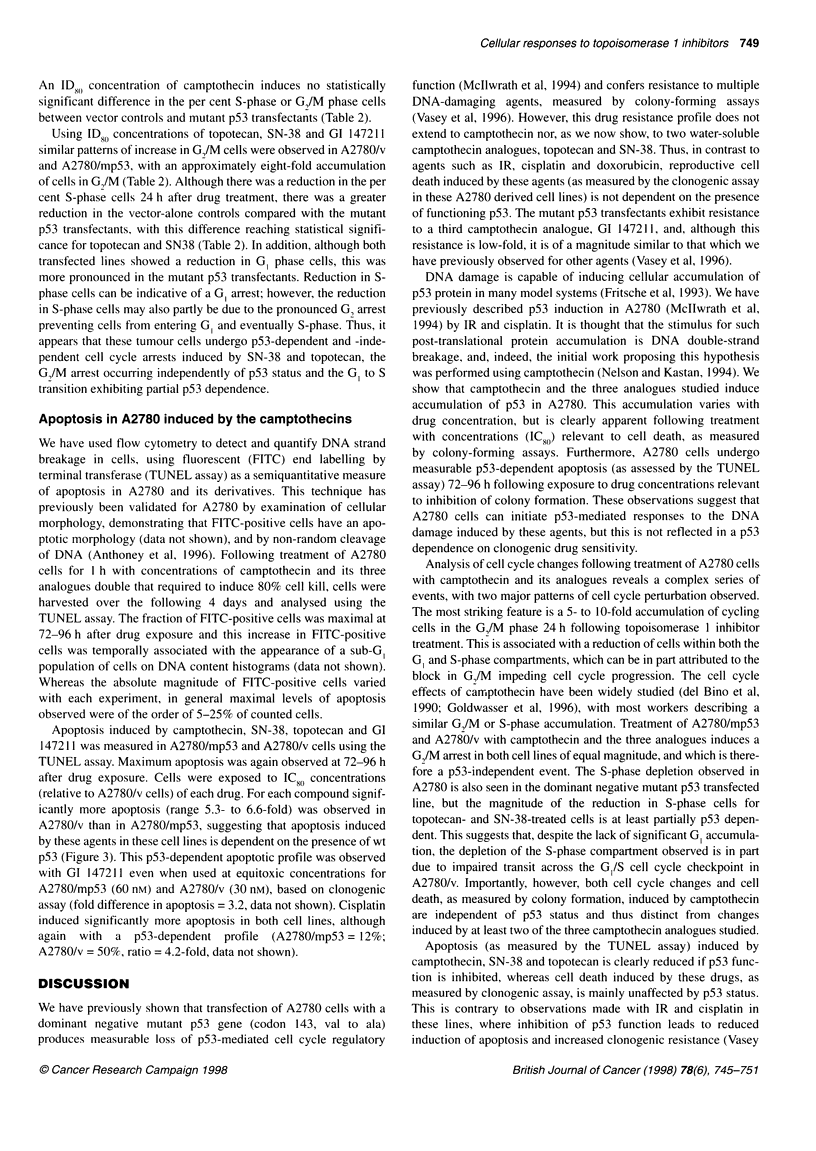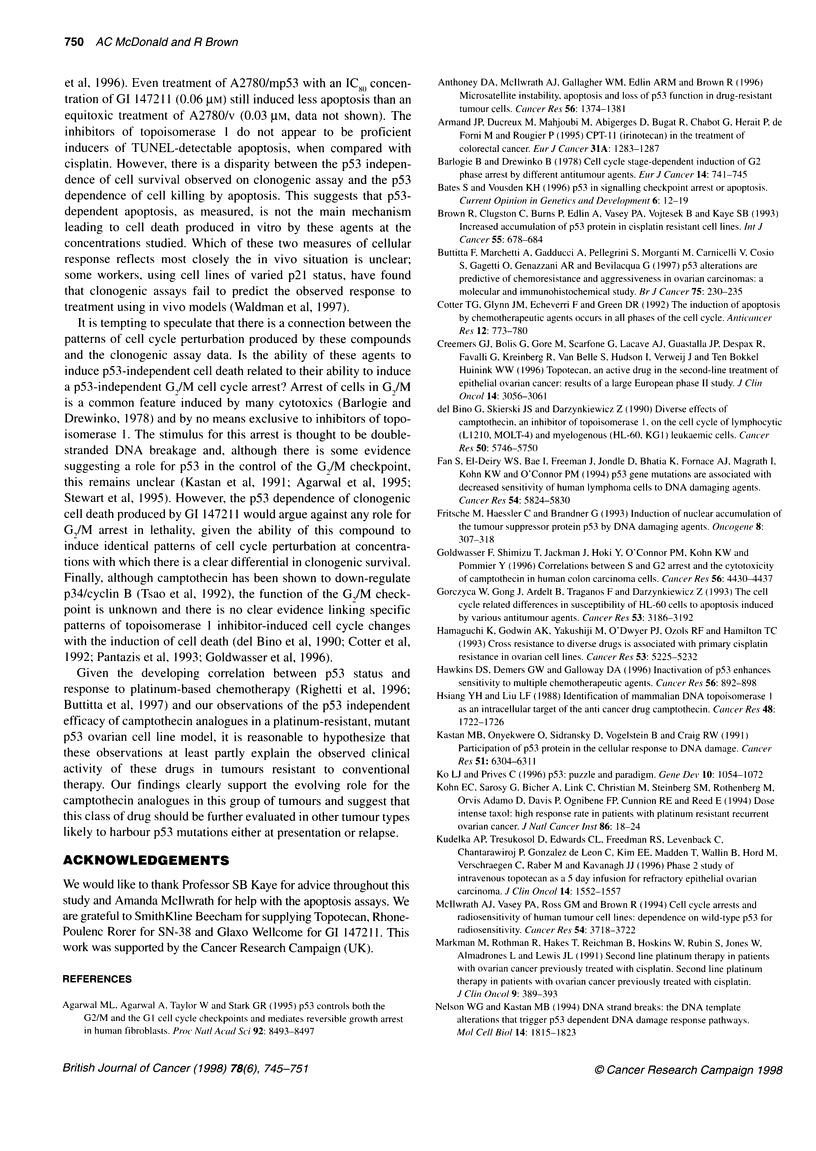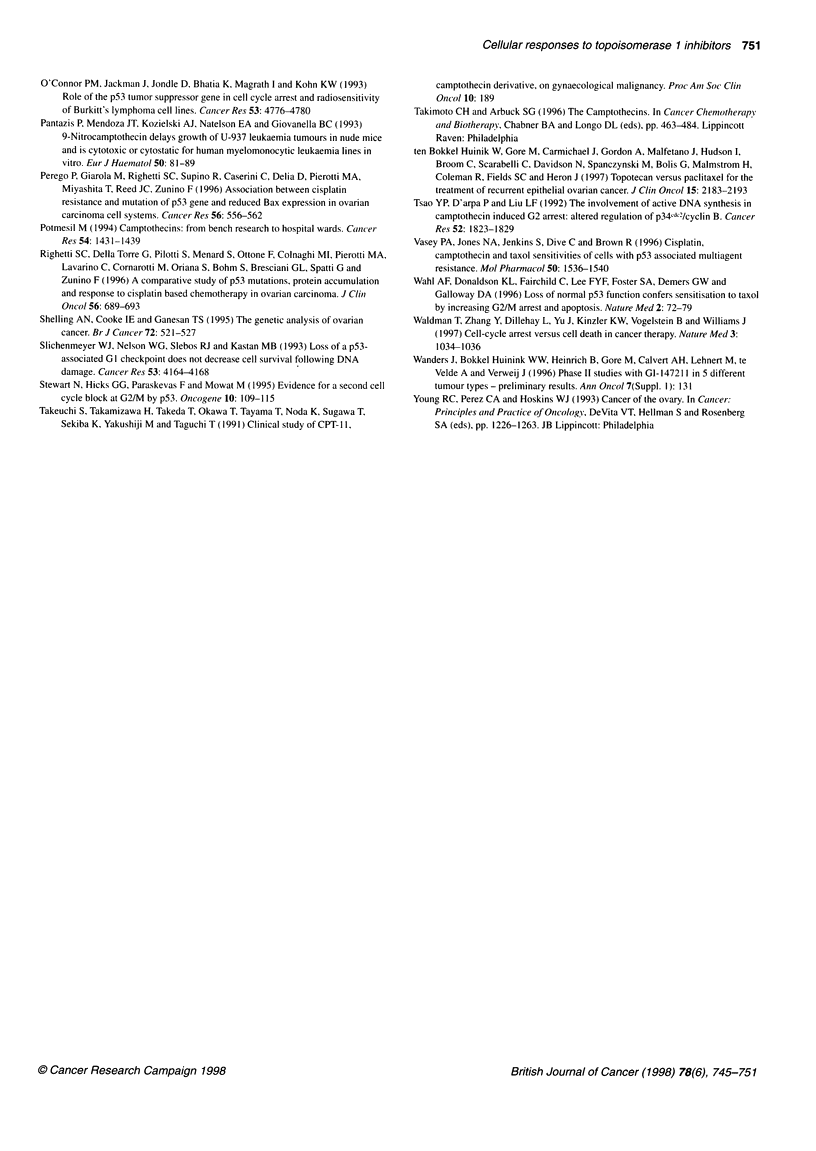# Induction of p53-dependent and p53-independent cellular responses by topoisomerase 1 inhibitors.

**DOI:** 10.1038/bjc.1998.571

**Published:** 1998-09

**Authors:** A. C. McDonald, R. Brown

**Affiliations:** CRC Department of Medical Oncology, CRC Beatson Laboratories, Glasgow, UK.

## Abstract

**Images:**


British Journal of Cancer (1998) 78(6), 745–751
© 1998 Cancer Research Campaign

### Induction of p53-dependent and p53-independent cellular responses by topoisomerase 1 inhibitors

**AC McDonald and R Brown**

CRC Department of Medical Oncology, CRC Beatson Laboratories, Garscube Estate, Switchback Road, Glasgow G61 1BD, UK

**Summary** We have previously shown that loss of p53 function in A2780 human ovarian adenocarcinoma cells confers increased clonogenic resistance to several DNA-damaging agents, but not to taxol or camptothecin. We have now extended these studies, comparing wild-type p53-expressing A2780 cells with isogenic derivatives transfected with a dominant negative mutant (143; val to ala) p53. We show that, as well as retaining equivalent clonogenic sensitivity to camptothecin, mutant p53 transfectants of A2780 cells do not acquire significantly increased resistance to the camptothecin analogues topotecan and SN-38, the active metabolite of CPT-11. Compared with vector-alone transfectants they are, however, relatively (2.2-fold) resistant to GI 147211, a further camptothecin analogue undergoing clinical trial. Treatment of A2780 with camptothecin and each analogue produces an increase, maximal at 24–48 h after drug exposure, of cells in the $G_2$/M phase of the cell cycle and a decrease in both $G_1$ and S-phase cells. The $G_2$ arrest is independent of p53 function for camptothecin and the three analogues. All four compounds can induce apoptosis in A2780, which is reduced in mutant p53 transfectants, as measured using the terminal DNA transferase-mediated b-d UTP nick end labelling (TUNEL) assay. Thus, although p53-dependent apoptosis is induced by camptothecin, topotecan and SN-38 in this human ovarian carcinoma cell line, these drugs induce p53-independent death, as measured by clonogenic assay.

**Keywords:** camptothecin; apoptosis; cell cycle; p53; drug resistance

Despite high rates of response to initial platinum-based chemotherapy, 5-year survival rates among patients with advanced ovarian cancer are poor (Young et al, 1993). Early disease recurrence in initially responsive patients is associated with resistance to further platinum therapy (Markman et al, 1991) and there is a need for novel agents with activity in platinum-resistant and -refractory disease. The taxoids have activity in disease recurring after platinum therapy (Kohn et al, 1994). A further group of drugs also active in recurrent and platinum-resistant disease are analogues of camptothecin (Takeuchi et al, 1991; Kudelka et al, 1996; Wanders et al, 1996).

Although clinical trials of camptothecin sodium were abandoned because of toxicity (Takimoto and Arbuck, 1996), interest in this class of drug was renewed following the identification of its mechanism of action as an inhibitor of topoisomerase 1 (Hsiang and Liu, 1988) and the development of water-soluble analogues with improved toxicity profiles (Potmesil, 1994). Currently, two compounds are in advanced development: topotecan has activity in several tumour types and is licensed for use in platinum-refractory ovarian cancer (Creemers et al, 1996); irinotecan or CPT-11 is also active in a number of diseases, most notably large bowel cancers (Armand et al, 1995). The latter is a pro-drug, converted by carboxylesterase to the active metabolite SN-38. A third compound, analogous to topotecan but with enhanced in vitro activity, is GI 147211 (GG211), currently undergoing phase 2 evaluation (Wanders et al, 1996).

*Received 8 October 1997*
*Revised 9 February 1998*
*Accepted 19 February 1998*

*Correspondence to:* R Brown

Mutations of the p53 gene on chromosome 17 are one of the commonest genetic abnormalities found in human solid tumours, with a mutation frequency of 30–50% in ovarian cancer (Shelling et al, 1995). The role of p53 in determining tumour chemosensitivity remains controversial. Recently two groups examining both p53 immunostaining and p53 mutations have shown clinical correlations between normal p53 protein expression or wild-type (wt) *p53* status and tumour sensitivity to initial cisplatin-based chemotherapy (Righetti et al, 1996; Buttitta et al, 1997), supporting previous in vitro observations that loss of p53 function may lead to increased drug resistance in ovarian tumour cells (Brown et al, 1993; Perego et al, 1996). p53 is intimately involved in the cellular response to DNA damage, being central to two specific responses, the $G_1$/S cell cycle checkpoint and cell death via apoptosis (Bates and Vousden, 1996). Alteration in p53 status is associated with changes in the cellular sensitivity to DNA-damaging agents; however, in different tumour cell types the effects of loss of wild-type p53 function on chemosensitivity are conflicting, with increased sensitivity (Brown et al, 1993; Hawkins et al, 1996; Wahl et al, 1996), increased resistance (O'Connor et al, 1993; Fan et al, 1994; McIlwrath et al, 1994; Vasey et al, 1996) or unchanged sensitivity (Slichenmeyer et al, 1993) varyingly observed.

The A2780 human ovarian cancer cell line, derived from a chemo-naive patient, is sensitive to various DNA-damaging agents (Hamaguchi et al, 1993). It has functional, wt p53 and undergoes $G_1$ arrest following treatment with ionizing radiation (IR) (Brown et al, 1993). Transfection of a dominant negative mutant of p53 into A2780 cells causes loss of p53 function as evidenced by abrogation of an IR-induced $G_1$ arrest (McIlwrath et al, 1994). The central role of p53 function in determining sensitivity of these cells to DNA damage is demonstrated by the

**Table 1** Relative sensitivity of A2780/mp53 and A2780/v to camptothecin and analogues

| | A2780/mp53 ($IC_{50}$) | A2780/v ($IC_{50}$) | Relative resistance | A2780/mp53 ($IC_{80}$) | A2780/v ($IC_{80}$) | Relative resistance |
|---|---|---|---|---|---|---|
| Doxorubicin[a] | 70 nM | 27 nM | 2.6-fold | 140 nM | 50 nM | 2.8 |
| Cisplatin[a] | 8 μM | 2.5 μM | 3.2-fold | 16 μM | 6.5 μM | 2.5 |
| Camptothecin | 59 nM | 54 nM | 1.2-fold | 116 nM | 104 nM | 1.1 |
| Topotecan | 200 nM | 150 nM | 1.3-fold | 420 nM | 280 nM | 1.5 |
| SN-38 | 40 nM | 30 nM | 1.4-fold | 78 nM | 58 nM | 1.3 |
| GI 147211 | 34 nM | 15 nM | 2.3-fold | 66 nM | 30 nM | 2.2 |

Resistance factors are shown for $IC_{50}$ and $IC_{80}$ drug concentrations and are derived from regression analysis of survival curves. [a]The data for cisplatin and doxorubicin are from Vasey et al (1996).

**Figure 1** Induction of p53 protein in A2780 cell line following treatment with camptothecin, GI 147211, SN-38 and topotecan assessed by Western immunoblotting. All extracts made 24 h following a 1-h drug exposure. Lane 1 in each blot are extracts from untreated controls; lanes 2–6, extracts made following drug exposure, with concentrations increasing fivefold in sequential lanes. Camptothecin: lane 2, 9.6 nM; lane 3, 0.05 μM; lane 4, 0.24 μM; lane 5, 1.2 μM; lane 6, 6 μM; GI 147211: lane 2, 1.6 nM; lane 3, 8 nM; lane 4, 0.04 μM; lane 5, 0.2 μM; lane 6 = 1 μM; SN-38: lane 2, 2.9 nM; lane 3, 14 nM; lane 4, 0.07 μM; lane 5, 0.35 μM; lane 6, 1.8 μM; topotecan: lane 2, 12 nM; lane 3, 0.06 μM; lane 4, 0.3 μM; lane 5, 1.5 μM; lane 6, 7.5 μM

increased clonogenic resistance of these mutant p53 transfectants to several anti-cancer agents, including IR, cisplatin and doxorubicin (Vasey et al 1996). However, this multiagent resistance profile does not extend to taxol or the topoisomerase 1 inhibitor camptothecin. Given this spectrum of drug resistance and the antitumour activity of topoisomerase 1 inhibitors in early clinical studies of pretreated and platinum-refractory ovarian cancer (Kudelka et al, 1996; ten Bokkel Huinik et al 1997), we have evaluated the in vitro effects of camptothecin and three water-soluble analogues within these model systems, specifically examining the effects of these agents on cellular accumulation of p53 protein, cell cycle alteration, clonogenic cell sensitivity and apoptosis.

### MATERIALS AND METHODS

### Cell lines

The following human cell lines were used: A2780, a human ovarian cancer cell line known to express only wt p53 gene sequences (Brown et al, 1993); A2780/mp53, an A2780 derivative transfected with mutant p53 (codon 143, val to ala); A2780/v, a further A2780 derivative transfected with vector alone (Brown et al, 1993). A2780/mp53 lacks a radiation-induced $G_1$ arrest, which is retained by A2780/v (McIlwrath et al, 1994). We have observed unstable expression of this phenotype and therefore regular confirmation of abrogation of $G_1$ arrest was performed, with cultures frequently regrown from frozen stocks. All cell lines were maintained as monolayers in RPMI-1640 medium with 10% fetal calf serum (FCS) at 37°C in 95% air/5% carbon dioxide. Continual positive selection in favour of expression of the linked geneticin resistance marker was performed for transfectant lines. All cell lines were free of *Mycoplasma* contamination.

### Drug sensitivity assays

Drug sensitivity was assessed using colony-forming assays; cells were seeded at $10^3$ into 10-$cm^2$ plates, a range of concentrations of relevant drug being added 24 h later, for an exposure period of 1 h. Assays were performed in triplicate. Statistical analysis was determined on six separate assays per line at a defined concentration of drug. After 10 days' incubation, colonies were stained with Giemsa (BDH) and those with more than 200 cells were counted. Surviving fractions were calculated as a percentage of colonies on drug-treated relative to untreated plates.

### Cell cycle analysis by flow cytometry

Proportions of cells in different phases of the cell cycle were assessed by incorporation of bromodeoxyuridine (BrdUrd),

 *© Cancer Research Campaign 1998*

**Figure 2** Cell cycle changes over 48 h following a 1-h exposure of A2780, A2780/mp53 and A2780/v to 0.1 μM camptothecin, an $IC_{80}$ concentration. Values (fraction) are expressed as a ratio relative to untreated controls (which are therefore represented as unity). Values plotted are the mean of at least four experiments, with error bars representing 1 standard deviation. **A** $G_1$-phase. **B** S-phase. **C** $G_2$ M-phase. —●—, A2780; - -■- -, A2780/mp53; - -▲- -, A2780/v propidium iodide (PI) staining and flow cytometric analysis as described previously (McIlwrath et al, 1994). Exponentially growing cells were plated at $5 \times 10^5$ per 10-$cm^2$ plate into medium and incubated for 3–4 days. Cells were then exposed to growth medium containing drug for 1 h. At various times after treatment, medium was replaced by medium containing 10 μM BrdUrd and cells incubated for 4 h. They were then harvested, fixed, washed with phosphate-buffered saline (PBS) and partially denatured in 2 N hydrochloric acid. The cells were incubated with an anti-BrdUrd mouse MAb (Dako); bound complexes were subsequently detected with goat anti-mouse fluoroscein isothiocyanate (Sigma), stained with PI and analysed using a fluorescence-assisted cell sorter (Becton Dickinson, San Jose, CA, USA). No marked difference in total cell number was noted between treated and control cultures.

### Detection of apoptosis by flow cytometry

Apoptotic cells were detected as described previously (Gorczyca et al, 1993; Anthoney et al, 1996). Exponentially growing cells were exposed to either drug-free or drug-containing medium for 1 h and harvested at various times thereafter. Attached and non-attached cells were collected and fixed firstly in 1% formaldehyde for 30 min and then 70% ethanol in PBS. Cells were rehydrated in PBS and aliquots of $10^6$ cells incubated sequentially with cacodylate buffer (0.2 M potassium cacodyalate, 2.5 mM Tris-HCl (pH 6.6), 2.5 mM cobalt chloride, 0.25 mg $ml^{-1}$ BSA, five units of terminal DNA transferase/$10^6$ cells and 0.5 nmol biotin-dUTP/$10^6$ cells) and 4 × SSC/0.1% Triton X-100 containing 5% Marvel and 5 μg $ml^{-1}$ fluorosceinated avidin before staining with PI. Cellular fluoresence was detected using a fluorescence-assisted cell sorter (Becton Dickinson).

### Immunoassays

Cell extracts were prepared for p53 and topoisomerase 1 immunoassay by lysing exponentially growing cells as described previously (McIlwrath et al, 1994). Protein concentrations were determined using the Biorad protein assay (Richmond, CA). Immunoblotting was performed as described previously (Brown et al, 1993), probed with p53 antibody, (pAB-6 Oncogene Science) or polyclonal antibody from scleroderma patient serum to topoisomerase 1 (Topogen Inc, Columbus, OH, USA). Blots were visualized with enhanced chemiluminescence (Amersham).

### RESULTS

### Clonogenic sensitivity of A2780/mp53 and A2780/v to camptothecin and analogues

We have previously shown that mutant p53 cDNA (codon 143 val to ala) transfected into A2780 cells acts as a dominant negative mutant and induces loss of an IR-induced $G_1$ arrest (McIlwrath et al, 1994). These A2780/mp53 transfectants exhibit increased resistance to multiple DNA-damaging agents, such as IR, cisplatin and doxorubicin, but retain sensitivity to camptothecin (Vasey et al, 1996). We have confirmed the lack of cross-resistance of A2780/mp53 to camptothecin and examined its sensitivity to the camptothecin analogues, topotecan, SN-38 and GI147211 (Table 1). At drug concentrations capable of reducing clonogenic survival to 50% and 80% of untreated controls ($IC_{50}/IC_{80}$), the mutant p53 transfectants were equivalently sensitive to camptothecin, relative

© Cancer Research Campaign 1998 

**Table 2** Cell cycle distribution of A2780/v and A2780/mp53 after drug treatment

| | Cells in $G_1$ phase (%) | | Cells in S-phase (%) | | Cells in $G_2$ phase (%) | |
|---|---|---|---|---|---|---|
| | A2780/v | A2780/mp53 | A2780/v | A2780/mp53 | A2780/v | A2780/mp53 |
| Untreated controls | 50.2 (1.6) | 49 (2.1) | 45.4 (3.2) | 46.4 (1.8) | 4.6 (0.8) | 4.4 (1.3) |
| Ionizing radiation | 69.2 (4.4) | 53.2 (1.9) | 15.8 (1.7) | 37.5 (1.8)* | 14.5 (3.8) | 9.8 (2.2) |
| Camptothecin | 40.6 (3.6) | 39.2 (5.8) | 24.6 (4.1) | 28.8 (2.8) | 33.6 (2.7) | 31 (4.5) |
| SN-38 | 37.6 (4.4) | 29 (2.3)* | 21.8 (2.5) | 31.4 (4.7)* | 39 (3.8) | 33.8 (2.8) |
| Topotecan | 39.2 (4.2) | 30.8 (2.9)* | 23.2 (2.3) | 32.6 (4.8)* | 35 (1.5) | 33.5 (2.6) |
| GI147211 | 39.6 (2.8) | 32.4 (2.9)* | 20.8 (1.8) | 21.8 (6.7) | 39 (3.8) | 33.8 (2.8) |

The percentage of cells in each phase of the cell cycle, as determined by 4-h pulse BrdUrd labelling and FACS analysis, 24 h after a 1-h exposure to $IC_{80}$ concentrations of each drug. Figures in brackets represent 1 standard deviation of the mean. *Statistically significant difference between A2780/v and A2780/mp53 ($P < 0.05$ using $t$-test).

**Figure 3** Per cent apoptotic cells in A2780/mp53 and A2780/v at 72 h after drug exposure, as measured by the TUNEL assay. Equitoxic ($IC_{80}$) drug concentrations used in each case (camptothecin, 0.1 μM; SN-38, 0.07 μM; topotecan, 0.3 μM) aside from GI147211 (concentration, 0.04 μM, $IC_{80}$ for A2780/v, but $IC_{50}$ for A2780/mp53). Error bars represent one standard deviation of the mean. □, A2780/mp53; ▥, A2780/v to vector-alone controls. Marginal resistance is observed to both topotecan and SN-38 with a more marked 2.2- to 2.3-fold resistance to GI 147211 noted. In order to allow statistical comparison, surviving fraction was compared between the lines at drug concentrations giving approximately 0.2 surviving fraction. Thus, at concentrations of 0.1 μM camptothecin, 0.05 μM SN-38 and 0.5 μM topotecan no significant difference in drug sensitivity is observed between A2780/v and A2780/mp53 (using a Student $t$-test). However, A2780/mp53 shows significant cross-resistance to 0.04 μM GI 147211 compared with A2780/v ($P < 0.01$).

### Induction of p53 protein accumulation in A2780

We have examined accumulation of p53 protein after treatment of A2780 cells with camptothecin and the water-soluble analogues topotecan, SN38 and GI 147211. Treating A2780 with a range of drug concentrations, from those causing no detectable effect on clonogenic survival to doses in excess of that required to produce 100% lethality, leads to p53 protein accumulation 24 h after treatment (Figure 1).

Concentrations of drug used in specific lanes are approximately equitoxic (lane 4 approximating to an $IC_{80}$ concentration) and the magnitude of p53 protein induction varies as a function of drug concentration. Similar patterns of induction are observed for camptothecin, SN-38 and topotecan; however, GI 147211 more readily induces p53 protein at lower drug concentrations. Treatment of A2780 with topoisomerase 1 inhibitors at a drug concentration known to cause 80% reduction in clonogenic survival ($IC_{80}$) (0.1 μM camptothecin, 0.3 μM topotecan, 0.07 μM SN-38 and 0.04 μM GI 147211) for 1 h induces accumulation of p53 protein within 4–8 hours of treatment, maximal at 24 h following drug exposure (data not shown). Thus, each of these topoisomerase 1 inhibitors induces comparable accumulation of p53 after treatment of A2780 cells.

The p53 protein is a transcriptional transactivator, capable of influencing expression of a variety of genes (Ko and Prives, 1996). As we wished to examine cellular responses to topoisomerase 1 inhibitors, it was important to ensure that alteration of p53 status did not effect levels of expression of topoisomerase 1 in A2780 cells. Cell extracts of A2780 cells transfected with mutant p53 (codon 143 val to ala: A2780/mp53) were compared with vector-alone controls (A2780/v) for expression of topoisomerase 1 protein by Western analysis. No difference in the levels of topoisomerase 1 was observed between these lines (data not shown), suggesting that topoisomerase 1 transcription is unaffected by changes in p53 status.

### Cell cycle perturbation induced by the camptothecins in A2780 and dominant negative mutant p53 transfectants

Treatment of A2780 with 0.1 μM camptothecin (an $ID_{80}$ concentration) produces a characteristic pattern of cell cycle changes over 48 h following exposure (Figure 2). Changes to the distribution of cells within the cell cycle are apparent 4 h after drug exposure, with an increase in cells in the $G_2$/M phase. This increase is maximal at 24 h, with a five- to tenfold accumulation of cells within $G_2$/M phase. Treatment with camptothecin also leads to a reduction in S-phase cells, again maximal at 24 h.

A2780 cells transfected with a dominant negative mutant of p53 lose an IR-induced $G_1$ arrest, whereas the $G_2$ arrest is unaffected (McIlwrath et al, 1994). As shown in Table 2, the per cent of S-phase cells in vector-alone transfectants of A2780 (A2780/v) is reduced from 45.4% to 15.8% after irradiation with 2-Gy γ-rays, with a concomitant increase in the percentage of $G_1$ cells. Mutant p53 transfectants (A2780/mp53) show markedly less reduction in per cent S-phase cells and little increase in the per cent $G_1$ cells.

© Cancer Research Campaign 1998

An $ID_{80}$ concentration of camptothecin induces no statistically significant difference in the per cent S-phase or $G_2$/M phase cells between vector controls and mutant p53 transfectants (Table 2).

Using $ID_{80}$ concentrations of topotecan, SN-38 and GI 147211 similar patterns of increase in $G_2$/M cells were observed in A2780/v and A2780/mp53, with an approximately eight-fold accumulation of cells in $G_2$/M (Table 2). Although there was a reduction in the per cent S-phase cells 24 h after drug treatment, there was a greater reduction in the vector-alone controls compared with the mutant p53 transfectants, with this difference reaching statistical significance for topotecan and SN38 (Table 2). In addition, although both transfected lines showed a reduction in $G_1$ phase cells, this was more pronounced in the mutant p53 transfectants. Reduction in S-phase cells can be indicative of a $G_1$ arrest; however, the reduction in S-phase cells may also partly be due to the pronounced $G_2$ arrest preventing cells from entering $G_1$ and eventually S-phase. Thus, it appears that these tumour cells undergo p53-dependent and -independent cell cycle arrests induced by SN-38 and topotecan, the $G_2$/M arrest occurring independently of p53 status and the $G_1$ to S transition exhibiting partial p53 dependence.

### Apoptosis in A2780 induced by the camptothecins

We have used flow cytometry to detect and quantify DNA strand breakage in cells, using fluorescent (FITC) end labelling by terminal transferase (TUNEL assay) as a semiquantitative measure of apoptosis in A2780 and its derivatives. This technique has previously been validated for A2780 by examination of cellular morphology, demonstrating that FITC-positive cells have an apoptotic morphology (data not shown), and by non-random cleavage of DNA (Anthoney et al, 1996). Following treatment of A2780 cells for 1 h with concentrations of camptothecin and its three analogues double that required to induce 80% cell kill, cells were harvested over the following 4 days and analysed using the TUNEL assay. The fraction of FITC-positive cells was maximal at 72–96 h after drug exposure and this increase in FITC-positive cells was temporally associated with the appearance of a sub-$G_1$ population of cells on DNA content histograms (data not shown). Whereas the absolute magnitude of FITC-positive cells varied with each experiment, in general maximal levels of apoptosis observed were of the order of 5–25% of counted cells.

Apoptosis induced by camptothecin, SN-38, topotecan and GI 147211 was measured in A2780/mp53 and A2780/v cells using the TUNEL assay. Maximum apoptosis was again observed at 72–96 h after drug exposure. Cells were exposed to $IC_{80}$ concentrations (relative to A2780/v cells) of each drug. For each compound significantly more apoptosis (range 5.3- to 6.6-fold) was observed in A2780/v than in A2780/mp53, suggesting that apoptosis induced by these agents in these cell lines is dependent on the presence of wt p53 (Figure 3). This p53-dependent apoptotic profile was observed with GI 147211 even when used at equitoxic concentrations for A2780/mp53 (60 nM) and A2780/v (30 nM), based on clonogenic assay (fold difference in apoptosis = 3.2, data not shown). Cisplatin induced significantly more apoptosis in both cell lines, although again with a p53-dependent profile (A2780/mp53 = 12%; A2780/v = 50%, ratio = 4.2-fold, data not shown).

### DISCUSSION

We have previously shown that transfection of A2780 cells with a dominant negative mutant p53 gene (codon 143, val to ala) produces measurable loss of p53-mediated cell cycle regulatory function (McIlwrath et al, 1994) and confers resistance to multiple DNA-damaging agents, measured by colony-forming assays (Vasey et al, 1996). However, this drug resistance profile does not extend to camptothecin nor, as we now show, to two water-soluble camptothecin analogues, topotecan and SN-38. Thus, in contrast to agents such as IR, cisplatin and doxorubicin, reproductive cell death induced by these agents (as measured by the clonogenic assay in these A2780 derived cell lines) is not dependent on the presence of functioning p53. The mutant p53 transfectants exhibit resistance to a third camptothecin analogue, GI 147211, and, although this resistance is low-fold, it is of a magnitude similar to that which we have previously observed for other agents (Vasey et al, 1996).

DNA damage is capable of inducing cellular accumulation of p53 protein in many model systems (Fritsche et al, 1993). We have previously described p53 induction in A2780 (McIlwrath et al, 1994) by IR and cisplatin. It is thought that the stimulus for such post-translational protein accumulation is DNA double-strand breakage, and, indeed, the initial work proposing this hypothesis was performed using camptothecin (Nelson and Kastan, 1994). We show that camptothecin and the three analogues studied induce accumulation of p53 in A2780. This accumulation varies with drug concentration, but is clearly apparent following treatment with concentrations ($IC_{80}$) relevant to cell death, as measured by colony-forming assays. Furthermore, A2780 cells undergo measurable p53-dependent apoptosis (as assessed by the TUNEL assay) 72–96 h following exposure to drug concentrations relevant to inhibition of colony formation. These observations suggest that A2780 cells can initiate p53-mediated responses to the DNA damage induced by these agents, but this is not reflected in a p53 dependence on clonogenic drug sensitivity.

Analysis of cell cycle changes following treatment of A2780 cells with camptothecin and its analogues reveals a complex series of events, with two major patterns of cell cycle perturbation observed. The most striking feature is a 5- to 10-fold accumulation of cycling cells in the $G_2$/M phase 24 h following topoisomerase 1 inhibitor treatment. This is associated with a reduction of cells within both the $G_1$ and S-phase compartments, which can be in part attributed to the block in $G_2$/M impeding cell cycle progression. The cell cycle effects of camptothecin have been widely studied (del Bino et al, 1990; Goldwasser et al, 1996), with most workers describing a similar $G_2$/M or S-phase accumulation. Treatment of A2780/mp53 and A2780/v with camptothecin and the three analogues induces a $G_2$/M arrest in both cell lines of equal magnitude, and which is therefore a p53-independent event. The S-phase depletion observed in A2780 is also seen in the dominant negative mutant p53 transfected line, but the magnitude of the reduction in S-phase cells for topotecan- and SN-38-treated cells is at least partially p53 dependent. This suggests that, despite the lack of significant $G_1$ accumulation, the depletion of the S-phase compartment observed is in part due to impaired transit across the $G_1$/S cell cycle checkpoint in A2780/v. Importantly, however, both cell cycle changes and cell death, as measured by colony formation, induced by camptothecin are independent of p53 status and thus distinct from changes induced by at least two of the three camptothecin analogues studied.

Apoptosis (as measured by the TUNEL assay) induced by camptothecin, SN-38 and topotecan is clearly reduced if p53 function is inhibited, whereas cell death induced by these drugs, as measured by clonogenic assay, is mainly unaffected by p53 status. This is contrary to observations made with IR and cisplatin in these lines, where inhibition of p53 function leads to reduced induction of apoptosis and increased clonogenic resistance (Vasey

© Cancer Research Campaign 1998

et al, 1996). Even treatment of A2780/mp53 with an $IC_{80}$ concentration of GI 147211 (0.06 μM) still induced less apoptosis than an equitoxic treatment of A2780/v (0.03 μM, data not shown). The inhibitors of topoisomerase 1 do not appear to be proficient inducers of TUNEL-detectable apoptosis, when compared with cisplatin. However, there is a disparity between the p53 independence of cell survival observed on clonogenic assay and the p53 dependence of cell killing by apoptosis. This suggests that p53-dependent apoptosis, as measured, is not the main mechanism leading to cell death produced in vitro by these agents at the concentrations studied. Which of these two measures of cellular response reflects most closely the in vivo situation is unclear; some workers, using cell lines of varied p21 status, have found that clonogenic assays fail to predict the observed response to treatment using in vivo models (Waldman et al, 1997).

It is tempting to speculate that there is a connection between the patterns of cell cycle perturbation produced by these compounds and the clonogenic assay data. Is the ability of these agents to induce p53-independent cell death related to their ability to induce a p53-independent $G_2$/M cell cycle arrest? Arrest of cells in $G_2$/M is a common feature induced by many cytotoxics (Barlogie and Drewinko, 1978) and by no means exclusive to inhibitors of topoisomerase 1. The stimulus for this arrest is thought to be double-stranded DNA breakage and, although there is some evidence suggesting a role for p53 in the control of the $G_2$/M checkpoint, this remains unclear (Kastan et al, 1991; Agarwal et al, 1995; Stewart et al, 1995). However, the p53 dependence of clonogenic cell death produced by GI 147211 would argue against any role for $G_2$/M arrest in lethality, given the ability of this compound to induce identical patterns of cell cycle perturbation at concentrations with which there is a clear differential in clonogenic survival. Finally, although camptothecin has been shown to down-regulate p34/cyclin B (Tsao et al, 1992), the function of the $G_2$/M checkpoint is unknown and there is no clear evidence linking specific patterns of topoisomerase 1 inhibitor-induced cell cycle changes with the induction of cell death (del Bino et al, 1990; Cotter et al, 1992; Pantazis et al, 1993; Goldwasser et al, 1996).

Given the developing correlation between p53 status and response to platinum-based chemotherapy (Righetti et al, 1996; Buttitta et al, 1997) and our observations of the p53 independent efficacy of camptothecin analogues in a platinum-resistant, mutant p53 ovarian cell line model, it is reasonable to hypothesize that these observations at least partly explain the observed clinical activity of these drugs in tumours resistant to conventional therapy. Our findings clearly support the evolving role for the camptothecin analogues in this group of tumours and suggest that this class of drug should be further evaluated in other tumour types likely to harbour p53 mutations either at presentation or relapse.

### ACKNOWLEDGEMENTS

We would like to thank Professor SB Kaye for advice throughout this study and Amanda McIlwrath for help with the apoptosis assays. We are grateful to SmithKline Beecham for supplying Topotecan, Rhone-Poulenc Rorer for SN-38 and Glaxo Wellcome for GI 147211. This work was supported by the Cancer Research Campaign (UK).

### REFERENCES

Agarwal ML, Agarwal A, Taylor W and Stark GR (1995) p53 controls both the G2/M and the G1 cell cycle checkpoints and mediates reversible growth arrest in human fibroblasts. *Proc Natl Acad Sci* **92**: 8493–8497

Anthoney DA, McIlwrath AJ, Gallagher WM, Edlin ARM and Brown R (1996) Microsatellite instability, apoptosis and loss of p53 function in drug-resistant tumour cells. *Cancer Res* **56**: 1374–1381

Armand JP, Ducreux M, Mahjoubi M, Abigerges D, Bugat R, Chabot G, Herait P, de Forni M and Rougier P (1995) CPT-11 (irinotecan) in the treatment of colorectal cancer. *Eur J Cancer* **31A**: 1283–1287

Barlogie B and Drewinko B (1978) Cell cycle stage-dependent induction of G2 phase arrest by different antitumour agents. *Eur J Cancer* **14**: 741–745

Bates S and Vousden KH (1996) p53 in signalling checkpoint arrest or apoptosis. *Current Opinion in Genetics and Development* **6**: 12–19

Brown R, Clugston C, Burns P, Edlin A, Vasey PA, Vojtesek B and Kaye SB (1993) Increased accumulation of p53 protein in cisplatin resistant cell lines. *Int J Cancer* **55**: 678–684

Buttitta F, Marchetti A, Gadducci A, Pellegrini S, Morganti M, Carnicelli V, Cosio S, Gagetti O, Genazzani AR and Bevilacqua G (1997) p53 alterations are predictive of chemoresistance and aggressiveness in ovarian carcinomas: a molecular and immunohistochemical study. *Br J Cancer* **75**: 230–235

Cotter TG, Glynn JM, Echeverri F and Green DR (1992) The induction of apoptosis by chemotherapeutic agents occurs in all phases of the cell cycle. *Anticancer Res* **12**: 773–780

Creemers GJ, Bolis G, Gore M, Scarfone G, Lacave AJ, Guastalla JP, Despax R, Favalli G, Kreinberg R, Van Belle S, Hudson I, Verweij J and Ten Bokkel Huinink WW (1996) Topotecan, an active drug in the second-line treatment of epithelial ovarian cancer: results of a large European phase II study. *J Clin Oncol* **14**: 3056–3061

del Bino G, Skierski JS and Darzynkiewicz Z (1990) Diverse effects of camptothecin, an inhibitor of topoisomerase 1, on the cell cycle of lymphocytic (L1210, MOLT-4) and myelogenous (HL-60, KG1) leukaemic cells. *Cancer Res* **50**: 5746–5750

Fan S, El-Deiry WS, Bae I, Freeman J, Jondle D, Bhatia K, Fornace AJ, Magrath I, Kohn KW and O'Connor PM (1994) p53 gene mutations are associated with decreased sensitivity of human lymphoma cells to DNA damaging agents. *Cancer Res* **54**: 5824–5830

Fritsche M, Haessler C and Brandner G (1993) Induction of nuclear accumulation of the tumour suppressor protein p53 by DNA damaging agents. *Oncogene* **8**: 307–318

Goldwasser F, Shimizu T, Jackman J, Hoki Y, O'Connor PM, Kohn KW and Pommier Y (1996) Correlations between S and G2 arrest and the cytotoxicity of camptothecin in human colon carcinoma cells. *Cancer Res* **56**: 4430–4437

Gorczyca W, Gong J, Ardelt B, Traganos F and Darzynkiewicz Z (1993) The cell cycle related differences in susceptibility of HL-60 cells to apoptosis induced by various antitumour agents. *Cancer Res* **53**: 3186–3192

Hamaguchi K, Godwin AK, Yakushiji M, O'Dwyer PJ, Ozols RF and Hamilton TC (1993) Cross resistance to diverse drugs is associated with primary cisplatin resistance in ovarian cell lines. *Cancer Res* **53**: 5225–5232

Hawkins DS, Demers GW and Galloway DA (1996) Inactivation of p53 enhances sensitivity to multiple chemotherapeutic agents. *Cancer Res* **56**: 892–898

Hsiang YH and Liu LF (1988) Identification of mammalian DNA topoisomerase 1 as an intracellular target of the anti cancer drug camptothecin. *Cancer Res* **48**: 1722–1726

Kastan MB, Onyekwere O, Sidransky D, Vogelstein B and Craig RW (1991) Participation of p53 protein in the cellular response to DNA damage. *Cancer Res* **51**: 6304–6311

Ko LJ and Prives C (1996) p53: puzzle and paradigm. *Gene Dev* **10**: 1054–1072

Kohn EC, Sarosy G, Bicher A, Link C, Christian M, Steinberg SM, Rothenberg M, Orvis Adamo D, Davis P, Ognibene FP, Cunnion RE and Reed E (1994) Dose intense taxol: high response rate in patients with platinum resistant recurrent ovarian cancer. *J Natl Cancer Inst* **86**: 18–24

Kudelka AP, Tresukosol D, Edwards CL, Freedman RS, Levenback C, Chantarawiroj P, Gonzalez de Leon C, Kim EE, Madden T, Wallin B, Hord M, Verschraegen C, Raber M and Kavanagh JJ (1996) Phase 2 study of intravenous topotecan as a 5 day infusion for refractory epithelial ovarian carcinoma. *J Clin Oncol* **14**: 1552–1557

McIlwrath AJ, Vasey PA, Ross GM and Brown R (1994) Cell cycle arrests and radiosensitivity of human tumour cell lines: dependence on wild-type p53 for radiosensitivity. *Cancer Res* **54**: 3718–3722

Markman M, Rothman R, Hakes T, Reichman B, Hoskins W, Rubin S, Jones W, Almadrones L and Lewis JL (1991) Second line platinum therapy in patients with ovarian cancer previously treated with cisplatin. Second line platinum therapy in patients with ovarian cancer previously treated with cisplatin. *J Clin Oncol* **9**: 389–393

Nelson WG and Kastan MB (1994) DNA strand breaks: the DNA template alterations that trigger p53 dependent DNA damage response pathways. *Mol Cell Biol* **14**: 1815–1823

 © Cancer Research Campaign 1998

O'Connor PM, Jackman J, Jondle D, Bhatia K, Magrath I and Kohn KW (1993) Role of the p53 tumor suppressor gene in cell cycle arrest and radiosensitivity of Burkitt's lymphoma cell lines. *Cancer Res* **53**: 4776–4780

Pantazis P, Mendoza JT, Kozielski AJ, Natelson EA and Giovanella BC (1993) 9-Nitrocamptothecin delays growth of U-937 leukaemia tumours in nude mice and is cytotoxic or cytostatic for human myelomonocytic leukaemia lines in vitro. *Eur J Haematol* **50**: 81–89

Perego P, Giarola M, Righetti SC, Supino R, Caserini C, Delia D, Pierotti MA, Miyashita T, Reed JC, Zunino F (1996) Association between cisplatin resistance and mutation of p53 gene and reduced Bax expression in ovarian carcinoma cell systems. *Cancer Res* **56**: 556–562

Potmesil M (1994) Camptothecins: from bench research to hospital wards. *Cancer Res* **54**: 1431–1439

Righetti SC, Della Torre G, Pilotti S, Menard S, Ottone F, Colnaghi MI, Pierotti MA, Lavarino C, Cornarotti M, Oriana S, Bohm S, Bresciani GL, Spatti G and Zunino F (1996) A comparative study of p53 mutations, protein accumulation and response to cisplatin based chemotherapy in ovarian carcinoma. *J Clin Oncol* **56**: 689–693

Shelling AN, Cooke IE and Ganesan TS (1995) The genetic analysis of ovarian cancer. *Br J Cancer* **72**: 521–527

Slichenmeyer WJ, Nelson WG, Slebos RJ and Kastan MB (1993) Loss of a p53-associated G1 checkpoint does not decrease cell survival following DNA damage. *Cancer Res* **53**: 4164–4168

Stewart N, Hicks GG, Paraskevas F and Mowat M (1995) Evidence for a second cell cycle block at G2/M by p53. *Oncogene* **10**: 109–115

Takeuchi S, Takamizawa H, Takeda T, Okawa T, Tayama T, Noda K, Sugawa T, Sekiba K, Yakushiji M and Taguchi T (1991) Clinical study of CPT-11, camptothecin derivative, on gynaecological malignancy. *Proc Am Soc Clin Oncol* **10**: 189

Takimoto CH and Arbuck SG (1996) The Camptothecins. In *Cancer Chemotherapy and Biotherapy*, Chabner BA and Longo DL (eds), pp. 463–484. Lippincott Raven: Philadelphia ten Bokkel Huinik W, Gore M, Carmichael J, Gordon A, Malfetano J, Hudson I, Broom C, Scarabelli C, Davidson N, Spanczynski M, Bolis G, Malmstrom H, Coleman R, Fields SC and Heron J (1997) Topotecan versus paclitaxel for the treatment of recurrent epithelial ovarian cancer. *J Clin Oncol* **15**: 2183–2193

Tsao YP, D'arpa P and Liu LF (1992) The involvement of active DNA synthesis in camptothecin induced G2 arrest: altered regulation of $p34^{cdc2}$/cyclin B. *Cancer Res* **52**: 1823–1829

Vasey PA, Jones NA, Jenkins S, Dive C and Brown R (1996) Cisplatin, camptothecin and taxol sensitivities of cells with p53 associated multiagent resistance. *Mol Pharmacol* **50**: 1536–1540

Wahl AF, Donaldson KL, Fairchild C, Lee FYF, Foster SA, Demers GW and Galloway DA (1996) Loss of normal p53 function confers sensitisation to taxol by increasing G2/M arrest and apoptosis. *Nature Med* **2**: 72–79

Waldman T, Zhang Y, Dillehay L, Yu J, Kinzler KW, Vogelstein B and Williams J (1997) Cell-cycle arrest versus cell death in cancer therapy. *Nature Med* **3**: 1034–1036

Wanders J, Bokkel Huinink WW, Heinrich B, Gore M, Calvert AH, Lehnert M, te Velde A and Verweij J (1996) Phase II studies with GI-147211 in 5 different tumour types – preliminary results. *Ann Oncol* **7**(Suppl. 1): 131

Young RC, Perez CA and Hoskins WJ (1993) Cancer of the ovary. In *Cancer: Principles and Practice of Oncology*, DeVita VT, Hellman S and Rosenberg SA (eds), pp. 1226–1263. JB Lippincott: Philadelphia

© Cancer Research Campaign 1998